# Detection of novel auto-antigens in patients with recurrent miscarriage: description of an approach and preliminary findings

**DOI:** 10.3325/cmj.2014.55.259

**Published:** 2014-06

**Authors:** Yuriy Kit, Marina Starykovych, Marie Vajrychova, Juraj Lenco, Danuta Zastavna, Rostyslav Stoika

**Affiliations:** 1 Institute of Cell Biology, National Academy of Sciences of Ukraine, Lviv, Ukraine; 2 Faculty of Military Health Sciences, Hradec Kralove, Czech Republic; 3 Institute of Hereditary Pathology, National Academy of Medical Sciences of Ukraine, Lviv, Ukraine

## Abstract

**Aim:**

To develop and test a protocol for isolation of potential auto-antigens from chorionic tissue that may be linked to recurrent miscarriage (RM).

**Methods:**

The strategy included: 1) isolation of IgGs tightly bound to chorionic tissue of RM patients by protein G chromatography; 2) construction of affinity columns using the chorionic antibodies for isolation of auto-antigens; 3) enrichment of auto-antigens from detergent extracted solution of chorionic proteins by affinity chromatography; 4) separation by dodecyl sulfate-electrophoresis followed by matrix-assisted laser desorption/ionization-time of flight mass spectrometry identification.

**Results:**

Five potential auto-antigens were detected: neutral alpha-glucosidase AB, endoplasmin, transitional endoplasmic reticulum ATPase, putative endoplasmin-like protein, and cytoplasmic actin 2.

**Conclusions:**

We developed a strategy for identification of auto-antigens in the chorionic tissue of women with RM, which could be of diagnostic and prognostic value.

Recurrent miscarriage (RM) is a spontaneous loss of three or more consecutive pregnancies with the same biological father during the first trimester of pregnancy. It affects 1%-2% of women and at least one half of the cases have no etiology (1,2). Overall, 75% of the affected women can be expected to have an unsuccessful subsequent pregnancy, although this rate falls in older women and women with an increasing number of miscarriages. A pivotal feature of RM is the response of auto-antibodies to different auto-antigens (3). Auto-antibodies toward laminin-1 (4) and GalNAcβ determinant of glycans have been detected in women with RM (5). Anti-phospholipid syndrome with anti-cardiolipin or lupus anticoagulant antibodies is present in 15% of women with the recurrent first and second trimester miscarriage (2,6). Sinсе the mаternаl immune response toward the fetus is associated with secondary infertility, it is important to search for novel auto-antigens that could contribute to the recurrent pregnancy losses. Although auto-antibodies have been proposed as an etiology of RM (2,5), the mechanisms leading to antibody development and targets of these auto-antibodies are poorly understood. Recently, we have detected higher levels of IgGs tightly bound to chorionic tissue of RM patients in comparison to findings from the embryonic kidney, lung, heart, intestine, and skin of a spontaneously aborted fetus due to other etiology (7). Thus, we investigated whether auto-antibodies possessing specificity to chorionic tissue proteins could allow detection of potential auto-antigens involved in the development of RM. The aim of this study was to develop an approach for detection and identification of auto-antigens in chorionic tissue of women with RM.

## Material and methods

### Patients

Chorionic tissue of 8 women (21-33 years old) with the history of spontaneous abortion (2 of them with 2 delivery losses and 6 with 3 consecutive miscarriages) with first trimester gestational age and blighted ovum were included in the study conducted during 2012 at the Institute of Hereditary Pathology, NAMS of Ukraine. Tissue was stored at -70°C. Blood serum of 3 women (27, 29, 33 years old) without obstetric and genetic history of diseases who had at least two healthy children was used as a control. The biological samples were collected and studied under the control of the Ethics Committee of the Institute of Hereditary Pathology, NAMS of Ukraine.

### Auto-antibodies purification

Samples were collected and carefully washed with phosphate buffered saline (PBS, pH 7.4) and homogenized in the Tris buffered saline (TBS, 20 mM Tris-HCl, pH 7.4) containing 1% Triton-X100 (TBS-T) in the presence of a mixture of protease inhibitors (Sigma-Aldrich, St. Louis, MO, USA). All steps were carried out at 4°C. The homogenates were incubated for 30 minutes at 4°C and centrifuged at 30 000 g, 4°C. Supernatants from those homogenates were loaded onto Protein G-Sepharose column (Sigma), sequentially washed with TBS-T, and then washed with TBS. ABs were eluted from the column with 0.1 M Gly-HCl buffer, pH 2.3, and immediately neutralized with 1.5 M Tris-HCl, pH 8.8. Protein concentration was measured by using the NanoDrop ND 1000 spectrophotometer (NanoDrop Technologies, Wilmington, DE, USA). Abs were biotynilated or used for preparation of the affinity matrix. As a control affinity matrix, IgGs purified from blood serum obtained from 3 healthy women by chromatography on Protein G-Sepharose column was used.

### Dodecyl sulfate-polyacrylamide gel electrophoresis (SDS-PAGE) and Western blot analysis

SDS electrophoresis of proteins was performed in 12% polyacrylamide as described previously (8). Proteins were electrically transferred onto nitrocellulose membrane, which was blocked by 5% non-fat milk in the PBS containing 0.05% Tween-20 at 20°C, 1 hour. In order to detect auto-antibodies in the chorionic detergent extracts, the blots were washed with PBS-Tween-20 three times for 5 minutes each, and then probed with secondary antibodies covalently bound to horseradish peroxidase (Sigma) dissolved in 5% non-fat milk/PBS-Tween-20 blocking solution. After incubation, the membrane was washed three times for 5 minutes in the PBS-Tween-20 and proteins were visualized by the ECL Western blotting detection reagents (Amersham, Little Chalfont, UK). To detect the auto-antibodies binding to chorionic proteins, they were first biotinylated according to the manufacturer’s protocol using hydrazine-biotin reagent (Sigma). The blots were incubated overnight at 4°С with biotinylated auto-antibodies (50 μg/mL). The membrane was incubated in the Avidin-HRP conjugates (Sigma), dissolved in 5% non-fat milk/PBS-Tween-20 blocking solution, and processed as described above.

### Preparation of the auto-antigens binding Sepharose (auto-antibodies Sepharose).

IgGs obtained from the chorionic tissue and IgGs obtained from blood serum of healthy donors were immobilized on the HC-Sepharose 4B (Sigma) according to the manufacturer’s protocol.

### Purification of auto-antigens

In order to purify the auto-antigens, Triton Х-100 extracted-proteins from the chorionic tissue of the RM patients were subjected to the affinity chromatography on the auto-antibodies Sepharose column. Protein extracts (3 mL, 6.3 mg/mL) were incubated with 1 mL of the auto-antibodies-matrix for 1 hour at 24°C in the TBS containing a mixture of protease inhibitors. After incubation, the auto-antibodies Sepharose were loaded onto a column and washed once in the TBS supplemented with 0.05% Twin 20 and three times with the TBS. As a control, Triton X-100-extracted proteins were subjected to chromatography on a column with Sepharose conjugated with IgG isolated from the blood serum of healthy human donors. Proteins were eluted from the affinity column with 0.1 M Gly-HCl buffer, pH 2.3, neutralized with 1.5 M Tris-HCl, pH 8.8, and separated by the SDS-PAGE electrophoresis in PAG gradient (7%-16.5%). Proteins on gels were stained with Coomassie G-250, and the appropriate protein bands were excised from gels, and used for protein identification by the matrix-assisted laser desorption/ionization-time of flight mass spectrometry (*MALDI-TOF*).

### MALDI-TOF analysis

Following electrophoresis, individual bands from the PAG were excised and treated by in-gel trypsin digestion. After proteolysis and extraction of the generated peptides, the mixture was analyzed using a 4800 MALDI-TOF/TOF mass spectrometer (AB Sciex, Foster City, CA, USA). Both tandem mass spectrometry (MS and MS/MS) spectra were searched in a combined search in GPS Explorer (AB Sciex, Framingham, MA, USA) using MASCOT engine (Matrix Science, London, UK) against human protein database downloaded from Universal Protein Resource (UniProt, *www.uniprot.org*).

## Results

**Figure 1:**
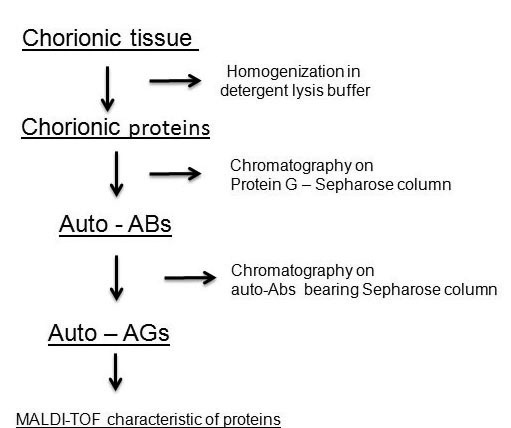
Scheme of affinity purification of the potential auto-antigens from chorionic tissue of recurrent miscarriage patients.

**Figure 2:**
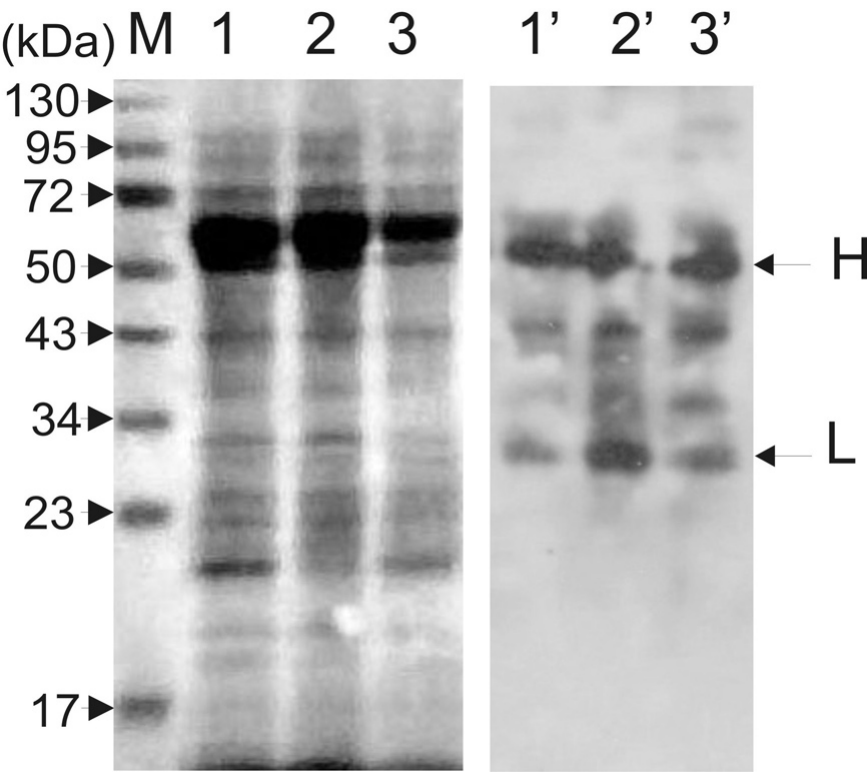
Determination of the presence of IgGs in typical preparations of triton X-100 extracted chorionic proteins by Western-blot analysis. The membrane was stained with Ponco C (lanes 1, 2, 3) and after washing was treated with mono specific horseradish peroxidase -conjugated rabbit anti-human IgGs (lanes 1’, 2’, 3′). Auto-antibodies presence was tested as described in Material and Methods.

**Figure 3:**
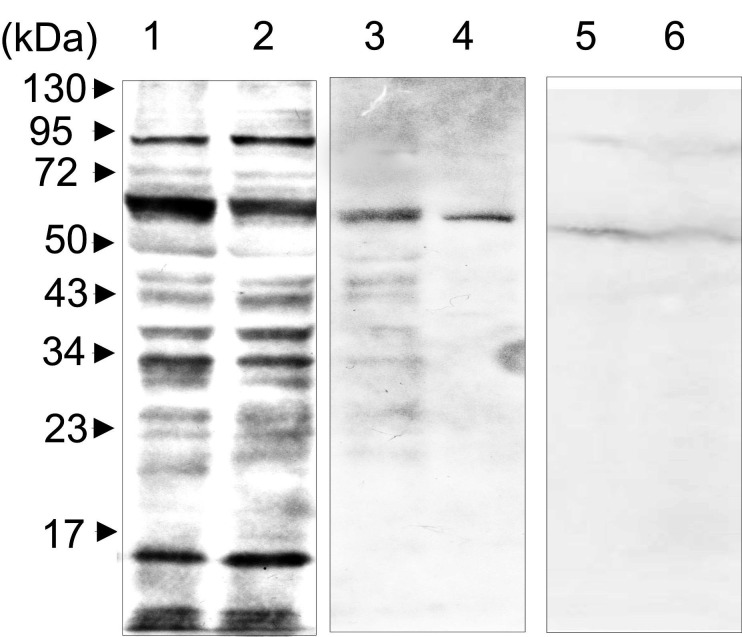
Western-blot analysis of typical preparations of Triton X-100 extracted chorionic proteins using biotinylated auto-IgGs isolated from chorionic tissues (lane 1, 2). As control we used IgGs isolated from blood serum of healthy donors (lanes 3,4) or Avidine-HRP reagent.

**Figure 4:**
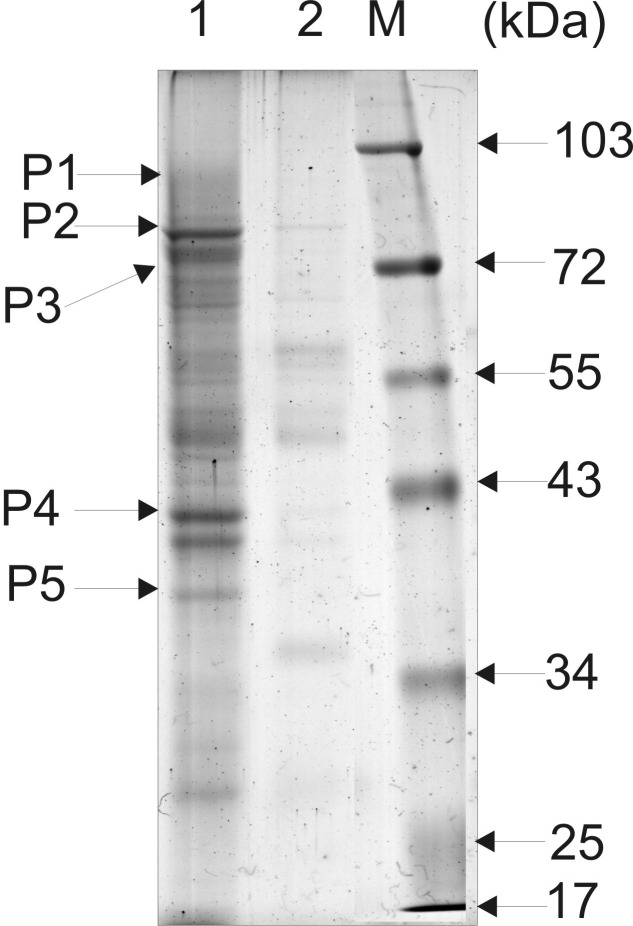
Dodecyl sulfate-electrophoresis in gradient of the polyacrylamide gel (7%-16%) in reducing condition of polypeptides isolated from chorionic extracts by affinity chromatography on auto-antibodies Sepharose column (lane 1) and IgG-antibodies healthy humans bearing Sepharose column (lane 2, control). M – the standards of molecular mass of proteins. On the left, polypeptides applied for matrix-assisted laser desorption/ionization mass spectrometry are shown.

We developed an approach to detect and identify potential auto-antigens that could be involved in the RM development (Figure 1). SDS-PAGE analysis of proteins of the chorionic tissue followed by their transfer to nitrocellulose membrane and probing with anti-human IgGs revealed heavy and light chains of human IgG in the lysates of the chorionic tissue (Figure 2). In order to isolate auto-antibodies from the chorionic tissue, its lysate was subjected to the affinity chromatography on the Protein G-Sepharose column. The IgGs fraction was eluted with acidic (pH 2.3) buffer allowing dissociation of various tightly bound immune complexes. A portion of the affinity isolated IgGs was used for biotinylation followed by western-blot analysis, while the remainder of the IgG fraction was coupled to Sepharose-matrix and used as an auto-antibodies bearing affinity sorbent. Before the affinity chromatography, the auto-antibodies were first assayed for their capacity to bind the chorionic tissue auto-antigens. Binding of the biotinylated auto-antibodies was compared with the binding of control biotinylated IgGs purified from blood serum of healthy human donors. The biotinylated antibodies eluted from chorionic tissue bound different proteins separated by the SDS-PAGE electrophoresis (Figure 3, lanes 1, 2). The biotinylated IgGs from blood serum of healthy donors, as well as the avidin-horseradish peroxidase conjugates bound only polypeptides migrating in the range of 67 kDa (Figure 3, lanes 3-6), while the biotinylated IgGs isolated from blood serum of RM women recognized a distinct set of chorionic proteins (Figure 4), which were subsequently identified by the MALDI-TOF MS as neutral alpha-glucosidase AB (107 kDa, Acc: ENPL_HUMAN), endoplasmin (92 kDa, Acc: GANAB_HUMAN), transitional endoplasmic reticulum ATPase (89 kDa, Acc: TERA_HUMAN), putative endoplasmin-like proteins (46 kDa, Acc: ENPLL_HUMAN), and cytoplasmic actin 2 (42 kDa, Acc: ACTG_HUMAN).

## Discussion

In this report, we described an approach that can be used for identification of the auto-antigens of chorionic tissue obtained from women with the RM. Application of this approach allowed us to identify 5 novel potential auto-antigens that could be associated with RM. Spontaneous pregnancy loss has been defined as 3 consecutive pregnancy losses prior to 20 weeks from the last menstrual period (2). However, there are no reliable data on the probability of RM in the population with 2 or 3 and more miscarriages, since the available data suggest that after 2 losses the risk of miscarriage in subsequent pregnancies is 30%, compared with 33% after 3 losses, which is not a big difference among patients without a history of a live birth (9). This strongly suggests the importance of evaluating patients with 2 losses, since such patients insist on their further clinical investigation. This is why we included in our study women with both 2 (2 patients) and 3 (6 patients) delivery losses.

Among 5 novel potential auto-antigens that we found to be associated with the RM, there is a neutral alpha-glucosidase AB. This enzyme [EC 3.2.1.84] is encoded in humans by the *GANAB* gene (10), and is located in the endoplasmic reticulum. It catalyzes the hydrolysis of the inner two α1,3-linked glucose residues present in all *N*-linked immature oligosaccharides. Another detected auto-antigen is a transitional endoplasmic reticulum ATPase [EC 3.6.4.6], also known as valosin-containing protein (VCP); it is an enzyme that is encoded in humans by the *VCP* gene (11). It participates in fragmentation of the Golgi stacks during mitosis and is necessary for their re-assembly after mitosis. It is also involved in the formation of the transitional endoplasmic reticulum. Our attempts to find in available databases the linkage between an appearance of autoantibodies to these enzymes and specific human autoimmune diseases or reproductive dysfunctions in women have failed. A link between actin autoantibodies and autoimmune diseases is well known, and it was found in patients with autoimmune liver diseases (12,13), celiac disease (14), and rheumatoid arthritis (15). The endoplasmin also known as Heat Shock Protein 90 kDa Beta Member 1 may participate in immune response in RM patients. Endoplasmin is an abundant molecular chaperone resident in the endoplasmic reticulum, and it plays a critical role in protein folding in the secretory pathway of Toll-like receptors and integrins (16,17). It has also been found to be an essential immune chaperone involved in regulation of both innate and adaptive immunity (18,19). Interestingly, HSP90B1 can serve as an endogenous activator for the dendritic cells. Besides, there are data that anti-HSP90B1 auto-antibodies could be directly engaged in reproductive dysfunction in women (20,21). Further studies are needed to investigate whether the appearance of anti-HSP90B1 auto-antibodies in blood serum of RM patients is linked to the overexpression of this stress-responsive protein in human chorionic tissues.

The application of the protocol described in our study allowed identification of potential auto-antigens in the chorionic tissue of women with RM. We hypothesize that the identified auto-antigens in chorionic tissue and the relevant auto-antibodies of blood serum contribute to RM, and thus could have diagnostic and prognostic value.

## References

[1] Rai R, Regan L (2006). Recurrent miscarriage.. Lancet.

[2] Baek KH, Lee EJ, Kim YS (2007). Recurrent pregnancy loss: the key potential mechanisms.. Trends Mol Med.

[3] Carp HJ, Selmi C, Shoenfeld Y (2012). The autoimmune bases of infertility and pregnancy loss.. J Autoimmun.

[4] Inagaki J, Kondo A, Lopezc L, Shoenfeld Y, Matsuura E (2004). Anti-laminin-1 autoantibodies, pregnancy loss and endometriosis.. Clin Dev Immunol.

[5] Blank M, Krause I, Dotan N, Anafi L, Eisenstein M, Cervera R (2012). Anti-GalNAcß: A novel anti-glycan autoantibody associated with pregnancy loss in women with antiphospholipid syndrome and in a mouse experimental model.. J Autoimmun.

[6] Meroni PL, Tedesco F, Locati M, Vecchi A, Di Simone N, Acaia B (2010). Anti-phospholipid antibody mediated fetal loss: still an open question from a pathogenic point of view.. Lupus.

[7] Starykovych M, Zastavna D, Juraj L, Stoika R, Kit Y. Anti- HSP90B1 autoantibodies as a novel potential marker for diagnostic of the recurrent miscarriage (RM). RECOOP annual project review meeting – 4th RECOOP TriNet meeting, October 10-13, 2013, Split, Croatia. RECOOP-HST Association; 2013.

[8] Laemmli UK (1970). Cleavage of structural proteins during the assembly of the head of bacteriophage T4.. Nature.

[9] Ford HB, Schutst DJ (2009). Recurrent pregnancy loss: etiology, diagnosis, and therapy.. Rev Obstet Gynecol..

[10] Martiniuk F, Smith M, Ellenbogen A, Desnick RJ, Astrin K, Mitra J (1983). Assignment of the gene for neutral alpha-glucosidase AB to chromosome 11.. Cytogenet Cell Genet.

[11] Pleasure IT, Black MM, Keen JH (1993). Valosin-containing protein, VCP, is a ubiquitous clathrin-binding protein.. Nature.

[12] Czaja AJ (2005). Autoantibodies in autoimmune liver disease.. Adv Clin Chem.

[13] Mackay IR (2011). A 50-year experience with autoimmune hepatitis: and where are we now?. J Gastroenterol.

[14] Porcelli B, Ferretti F, Vindinni C, Scapelloto C, Terzuoli L (2013). Detection of autoantibodies against actin filaments in celiac disease.. J Clin Lab Anal.

[15] Shrivastav M, Mittal B, Aggarwal A, Misra R (2002). Autoantibodies against cytoskeletal proteins in rheumatoid arthritis.. Clin Rheumatol.

[16] Maki RG, Old LJ, Srivastava PK (1990). Human homologue of murine tumor rejection antigen gp96: 5′-regulatory and coding regions and relationship to stress-induced proteins.. Proc Natl Acad Sci U S A.

[17] Schild H, Rammensee HG (2000). Gp96 – the immune system's Swiss army knife.. Nat Immunol.

[18] Liu B, Dai J, Zheng H, Stoilova D, Sun S, Li Z (2003). Cell surface expression of an endoplasmic reticulum resident heat shock protein gp96 triggers MyD88-dependent systemic autoimmune diseases.. Proc Natl Acad Sci U S A.

[19] Han JM, Kwon NH, Lee JY, Jeong SJ, Jung HJ, Kim HR (2010). Identification of gp96 as a novel target for treatment of autoimmune dsease in mice.. PLoS ONE.

[20] Choudhury A, Khole VV (2013). HSP90 antibodies: a detrimental factor responsible for ovarian dysfunction.. Am J Reprod Immunol.

[21] Pires ES, Choudhury AK, Idicula-Thomas S, Khole VV (2011). Anti-HSP90 autoantibodies in sera of infertile women identify a dominant, conserved epitope EP6 (380-389) of HSP90 beta protein.. Reprod Biol Endocrinol.

